# Application of next-generation sequencing for 24-chromosome aneuploidy screening of human preimplantation embryos

**DOI:** 10.1186/s13039-015-0143-6

**Published:** 2015-06-16

**Authors:** Haiyan Zheng, Hua Jin, Lian Liu, Jianqiao Liu, Wei-Hua Wang

**Affiliations:** Reproductive Medicine Center, Key Laboratory for Reproductive Medicine of Guangdong Province, Third Affiliated Hospital of Guangzhou Medical University, Guangzhou, China; Pacgenomics Inc, Agoura Hills, CA USA; Houston Fertility Laboratory, Vivere Health, Houston, TX USA

**Keywords:** Preimplantation genetic screening, Next-generation sequencing, Aneuploidy screening, Array comparative genomic hybridization, Blastocyst

## Abstract

**Background:**

Aneuploidy is a leading cause of repeat implantation failure and recurrent miscarriages. Preimplantation genetic screening (PGS) enables the assessment of the numeral and structural chromosomal errors of embryos before transfer in patients undergoing in vitro fertilization. Array comparative genomic hybridization (aCGH) has been demonstrated to be an accurate PGS method and in present thought to be the gold standard, but new technologies, such as next-generation sequencing (NGS), continue to emerge. Validation of the new comprehensive NGS-based 24-chromosome aneuploidy screening technology is still needed to determine the preclinical accuracy before it might be considered as an alternative method for human PGS.

**Results:**

In the present study, 43 human trophectoderm (TE) biopsy samples and 5 cytogenetically characterized cell lines (Coriell Cell Repositories) were tested. The same whole genome amplified product of each sample was blindly assessed with Veriseq NGS and Agilent aCGH to identify the aneuploidy status. The result showed that the NGS identified all abnormalities identified in aCGH including the numeral chromosomal abnormalities (again or loss) in the embryo samples and the structural (partial deletion and duplication) in the Coriell cell lines. Both technologies can identify a segmental imbalance as small as 1.8 Mb in size. Among the 41 TE samples with abnormal karyotypes in this study, eight (19.5 %) samples presented as multiple chromosome abnormalities. The abnormalities occurred to almost all chromosomes, except chromosome 6, 7, 17 and Y chromosome.

**Conclusions:**

Given its reliability and high level of consistency with an established aCGH methodology, NGS has demonstrated a robust high-throughput methodology ready for extensive clinical application in reproductive medicine, with potential advantages of reduced costs and enhanced precision. Then, a randomized controlled clinical trial confirming its clinical effectiveness is advisable to obtain a larger sequencing dataset and more evidence for the extensive use of NGS-based PGS.

## Background

Successful in vitro fertilization-embryo transfer (IVF-ET) is based partially on selection of viable embryos [1]. However, it is well known that many women fail to achieve a pregnancy even after transfer of good quality embryos. It had been suspected that a high incidence of chromosome aneuploidy in human oocytes and/or embryos might cause low implantation and pregnancy rates [2]. Aneuploidy is also a leading cause of miscarriages and congenital birth defects [3, 4]. The high frequency of aneuploidy during preimplantation development has led to the suggestion that embryos should be tested for chromosomal abnormalities before transfer to the uterus [5].

Assisted reproduction technology (ART) has incorporated genetic tools for genetic testing of preimplantation embryos, which is performed in patients with high risk for monogenic disorders [6] or chromosomal structural abnormalities [7]. Preimplantation genetic diagnosis (PGD) for aneuploidy screening of embryos derived from patients undergoing IVF, also termed preimplantation genetic screening (PGS), enables the assessment of the numeral and structural chromosomal constitution of embryos before transfer. It has been applied to treat patients with increased risk for aneuploid embryos, and then introduced into clinical practice to improve the chance of healthy conceptions after infertility treatment with poor prognoses, such as advanced maternal age, repeated implantation failure, and recurrent miscarriage [8, 9]. A latest research reviewed literatures on PGS for aneuploidy with analysis of all chromosomes showed that embryo implantation rates could be significantly increased by the transfer screened euploid embryos [10].

To improve ART success rates and reduce miscarriage rates, 24-chromosome copy number analysis, a test that is noninvasive, rapid, and sufficiently low cost for application to all patients, may be effective. The first molecular cytogenetic technique to be applied to interphase nuclei spread on slides was fluorescence in situ hybridization (FISH) with the use of specific probes for the chromosomes most commonly involved in aneuploidy. However, FISH-based PGS results were untenable by some reports [11, 12]. A large number of prospective randomized clinical trials (RCTs) have consistently failed to show any improvement in delivery rates with the use of FISH-based PGS [13], although a recent RCT has reported a significant increase in live birth rates in patients of advanced maternal age [14]. This was attributed to particularly the limited number of chromosomes analyzed. Therefore, the focus has now shifted to new technologies that allow all 24 chromosomes to be analyzed to provide a more accurate assessment of embryos.

Today, development of a range of molecular genetic technologies allows copy number analysis for all 24 chromosomes in single or small numbers of cells, such as biopsies from preimplantation embryos. A variety of methodologies for 24-chromosome analysis have been developed, including array comparative genomic hybridization (aCGH) [15], single-nucleotide polymorphism microarrays (SNP) [16], and quantitative polymerase chain reaction (Q-PCR) [17]. Array CGH was the first technology to be widely used for 24-chromosome copy number analysis [18] around the world despite the relatively high cost of testing multiple samples. This method uses microarray technology to deliver comprehensive aneuploidy screening through its ability to detect imbalances in any of the 24 chromosomes rather than the limited chromosome assessment achievable by FISH [15, 18]. Higher pregnancy and live birth rates than previously reported for FISH-based testing have been reported [15].

The latest advances in next-generation sequencing (NGS) methods are revolutionizing the way biological research is conducted and clinical diagnosis is performed. PGS is different from other clinical diagnosis, small amount of embryo cells, accurate data, simple data analysis, reliable instrument support, cost effectiveness and scalability are crucial factors to consider. Chromosomal copy number assessment based on NGS may offer several advantages to aCGH including reduced DNA sequencing cost, enhanced detection of partial or segmental aneuploidies as a result of the potential increase in chromosomal analysis resolution, the potential automation of the sequencing library preparation to minimize human errors, reduce hands-on time, and enable higher throughput and consistency [19–22].

In addition, the focus in the PGS field has now shifted from day 3 single blastomere biopsy to day 5/6 trophectoderm (TE) sampling and the use of comprehensive chromosome screening technologies, in order to provide a more accurate assessment of the reproductive potential of embryos. With the use of NGS with TE samples from blastocyst biopsies, both whole chromosome aneuploidy and segmental chromosome imbalances could be detected [21].

Potential improvements have been reported in human ART with the transfer of embryos examined with current comprehensive aneuploidy screening methods [19, 23–25]. However, application of the new comprehensive technologies is still needed to determine the preclinical accuracy before they might be considered within the standard of care in reproductive medicine. The present study investigated the accuracy of NGS technology for comprehensive chromosome screening as a preclinical step before its clinical application in the diagnosis of chromosomal aneuploidy on embryos at blastocyst stage.

## Results

### Workflows of NGS-based PGS and aCGH-based PGS

Workflows of NGS-based PGS and aCGH-based PGS are summarized in Table 1. As shown in Table 1, the procedures for embryo biopsy and sample collection are similar between aCGH-based PGS and NGS-based PGS except that a reference genomic DNA (gDNA) sample is necessary for aCGH. After sample collection, WGA is necessary for both aCGH and NGS, and it took three hours to process WGA. After WGA, the procedures are totally different between two technologies. As shown in Table 1, one of the most time-consuming procedures for aCGH is DNA hybridization to array slides. This process has been significantly improved and the time has been reduced from previous ~15 hrs to current 2 hrs. This improvement has made aCGH-based PGS can be done within 8 hrs. However, for NGS-based PGS, the most time-consuming procedure is DNA sequencing. Although DNA sequencing time has been dramatically reduced during the past few years, it still needs ~6 hrs to complete the DNA sequencing for current PGS purpose. Due to this reason, the procedures for NGS-based PGS can be done in ~14 hrs.

**Table 1:** Comparison of workflows using NGS and aCGH for 24-chromosome copy number analysis

	aCGH		NGS
1	Embryo biopsy	NA	Embryo biopsy
2	Sample collection	Reference gDNA (+10 min)	Sample collection
3	WGA (3 hrs)	WGA (3 hrs)	WGA (3 hrs)
4	Labeling of amplified DNA (1 hrs)	Pooling and labeling of amplified DNA (1 hrs)	Qualification and dsDNA input dilution (20 min)
5	Preparation of labeled DNA (1 hrs)	Preparation of labeled DNA (1 hrs)	Tagmentation (20 min)
6	Hybridization to array (2 hrs)		PCR amplification (50 min)
7	Washing and Scanning (20 min)		PCR clean-up (30 min)
8	Cytogenomics analysis (40 min)		Library normalization (30 min)
9	NA		Library pooling and loading (10 min)
10	N/A		Sequencing (6 hrs)
11	N/A		Bioinformatics analysis (2 hrs)
Total hrs	~8 hrs		~14 hrs

*NGS*: next generation sequencing

*aCGH*: array comparative genomic hybridization

*WGA*: whole-genome amplification

*NA*: not applicable

### Consistency of aneuploidy screening with NGS and aCGH

**Fig. 1:**
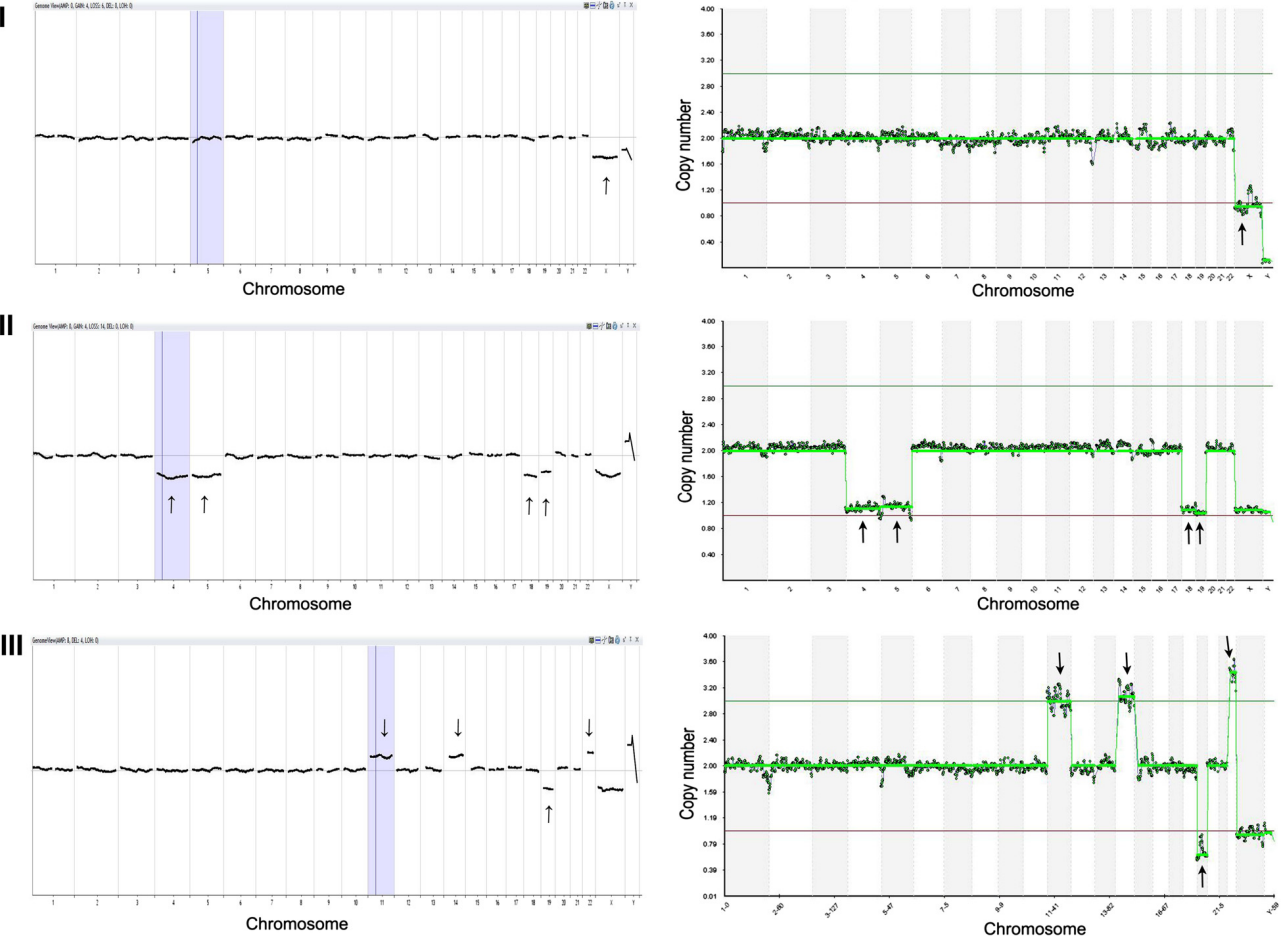
Representation samples of copy number changes observed in samples biopsied from blastocysts. Left panel: PGS results from array comparative genomic hybridization (aCGH) analysis; right panel: PGS results from next generation sequencing (NGS) analysis. X-axis indicate chromosome numbers (1-22, X and Y) and y-axis indicate chromosome copy number assignments (0, 1, 2, 3, or 4). The gains (copy number state >2) and losses (copy number state <2) of chromosomes in the right panel obtained with NGS exactly match those in the left panel obtained with aCGH. (I) Top left and right charts show a monosomy X from sample #3 in Table 2. (II) Middle left and right charts show monosomy 4, 5, 18 and 19 from sample #9 in Table 2. (III) Bottom left and right charts show trisomy 11, 14, 22 and monosomy19 from sample #35 in Table 2. Arrows indicate the locations of abnormal chromosomes

To test the feasibility of using NGS for PGS, a total of 43 TE samples biopsied from human blastocysts were tested. Successful results were obtained by NGS in all samples (100 %) included in the experiment. As showed in Table 2, when the NGS and aCGH aneuploidy results were compared, it was found that the NGS identified all abnormalities identified in aCGH. The predictive value of the NGS-based 24-chromosome aneuploidy screening protocol was 100 % for a normal (2/2) and 100 % for abnormal (41/41) index results. There were no false negative diagnoses for aneuploid chromosomes or embryos, or inaccurate predictions of gender. Comparative graph examples of NGS and aCGH results are shown in Fig. 1, in which samples with monosomy and trisomy were exhibited. All abnormal samples showed balanced, structural abnormalities, i.e. gain or loss of entire chromosomes.

**Table 2:** Chromosomes results of trophectoderm cells detected by next generation sequencing and array comparative genomic hybridization based preimplantation genetic screening

Samples	NGS karyotype	Array karyotype	Detected
1	47, XY, +15	arr (15) × 3	Yes
2	47, XY, +4	arr (4) × 3	Yes
3	45, XO	arr (X) × 1	Yes
4	45, XY, -22	arr (22) × 1	Yes
5	47, XXY	arr (X) × 2, (Y) × 1	Yes
6	48, XXY, +15	arr (X) × 2, (Y) × 1, (15) × 3	Yes
7	47, XX, +22	arr (22) × 3	Yes
8	45, XY, -21	arr (21) × 1	Yes
9	42, XY, -4, -5, -18, -19	arr (4) × 1, (5) × 1, (18) × 1,(19) × 1	Yes
10	47, XY, +15	arr (15) × 3	Yes
11	47, XY, +20	arr (20) × 3	Yes
12	47, XY, +16	arr (16) × 3	Yes
13	47, XY, +22	arr (22) × 3	Yes
14	47, XXY	arr (X) × 2, (Y) × 1	Yes
15	47, XX, +13	arr (13) × 3	Yes
16	45, XY, -10	arr (10) × 1	Yes
17	47, XY, +5	arr (5) × 3	Yes
18	47, XY, +14	arr (14) × 3	Yes
19	45, XY, -8	arr (8) × 1	Yes
20	45, XO	arr (X) × 1	Yes
21	45, XO	arr (X) × 1	Yes
22	45, XX, -16	arr (16) × 1	Yes
23	45, XX, -21	arr (21) × 1	Yes
24	45, XX, -19	arr (19) × 1	Yes
25	45, XY, -3	arr (3) × 1	Yes
26	45, XY, -22	arr (22) × 1	Yes
27	45, XY, -4	arr (4) × 1	Yes
28	46, XX	arr (1-22,X) × 2	Yes
29	47, XX, +19	arr (19) × 3	Yes
30	46, XY	arr (1-22) × 2, (XY) × 1	Yes
31	47, XY, +3	arr (3) × 3	Yes
32	45, XY, -1	arr (1) × 1	Yes
33	46, XY, +15, -16	arr (15) × 3, (16) × 1	Yes
34	46, XY, +21, +22	arr (21) × 3, (22) × 3	Yes
35	48, XY, +11, +14, -19, +22	arr (11) × 3, (14) × 3, (22) × 3, (19) × 1	Yes
36	47, XY, +10	arr (10) × 3	Yes
37	45, XX, -2	arr (2) × 1	Yes
38	46, XY, +4, -19	arr (4) × 3, (19) × 1	Yes
39	45, XY, -16	arr (16) × 1	Yes
40	48, XY, +19, +22	arr (19) × 3, (22) × 3	Yes
41	46, XY, +12, -14	arr (12) × 3, (14) × 1	Yes
42	45, XX, -9	arr (9) × 1	Yes
43	44, XX, -21, -22	arr (21) × 1, (22) × 1	Yes

### Segmental imbalance analysis with NGS and aCGH

**Fig. 2:**
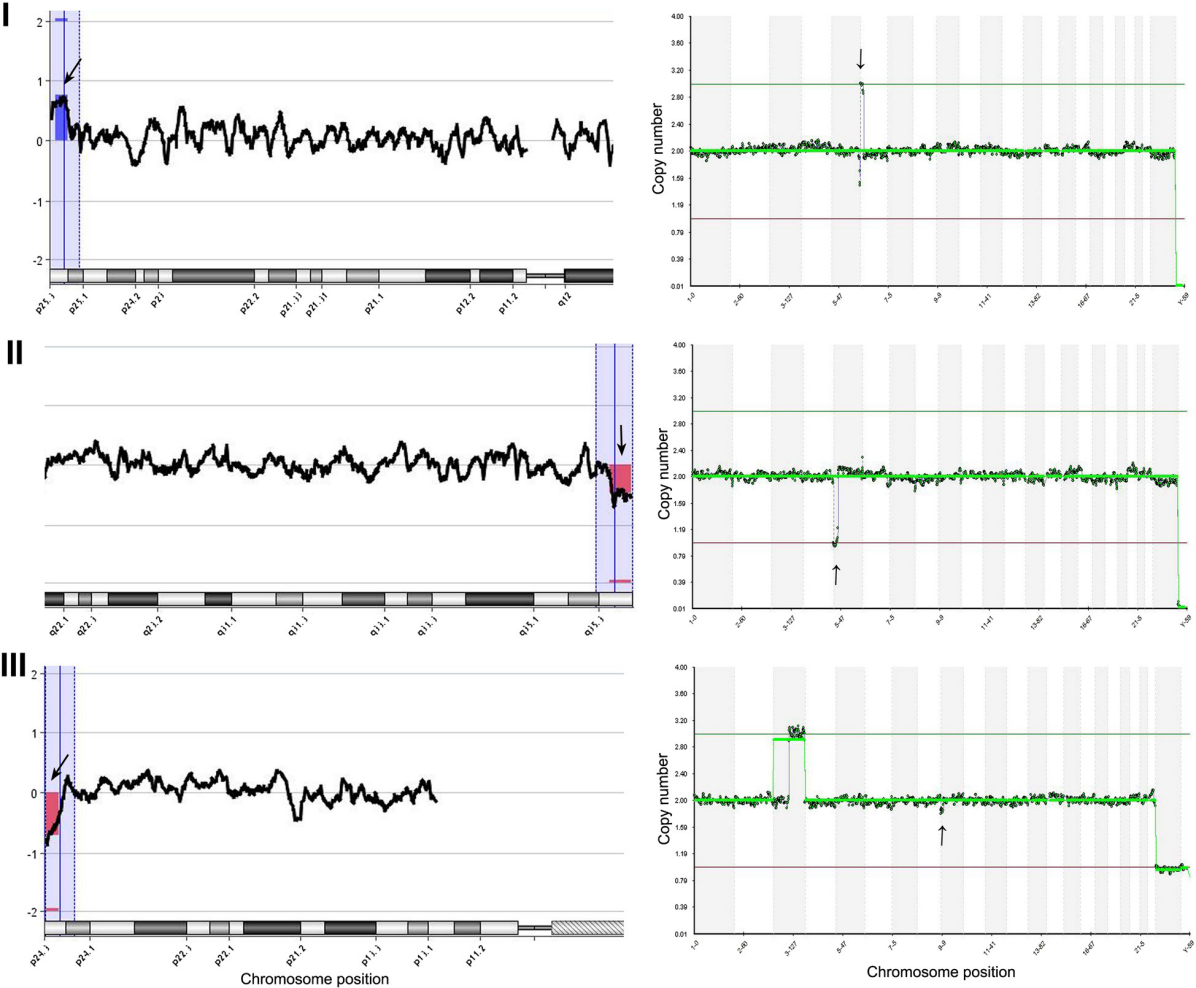
Examples of segmental imbalance detection selected from Coriell cell lines by next-generation sequencing (right panel) compared with array comparative genomic hybridization (left panel). Arrows in the right panel indicate locations of partial chromosomal imbalances. Blue and red boxes (indicated by arrows) in the left panel indicate same partial chromosomal imbalances (duplication or deletion), respectively. Only abnormal chromosomal segment (not all 24 chromosomes) are showed in the left panel. (I) Top left and right charts show a sample with a 2.19-Mb segmental duplication on the short arm of chromosome 6 from sample #5 in Table 3. (II) Middle left and right charts show a sample with a 2.53-Mb segmental deletion on the long arm of chromosome 5 from sample #2 in Table 3. (III) Bottom left and right charts show a sample with a 1.81-Mb segmental deletion on the short arm of chromosome 9 from sample #3 in Table 3

In order to further examine partial (imbalanced) chromosome abnormalities, we further tested 5 cytogenetically characterized cell lines with both aCGH and NGS. These cells lines have known chromosomal segmental breakpoints and sizes, and have been used for validation of different cytogenetic protocols. As shown in Table 3, NGS method identified the same microdeletions and amplifications of 5 Coriell cell lines as aCGH method. The sizes of the segmental errors were from 1.19 Mb to 3.89 Mb in the present study. As shown in Table 3 and Fig. 2, the smallest segment detected by both methods was 1.81 Mb. A sample (second chromosomal error in the sample #5) with 1.19 Mb microdeletion was not detected by both methods. The detailed breakpoints and sizes of segments in the chromosomes from each cell line were also showed in Table 3.

**Table 3:** Chromosomes results of Coriell cell lines with segmental imbalances

Samples	NGS karyotype	aCGH karyotype	Breakpoints	Size
1	47, XX, Dup (16) (p13.3)	47, XX, Dup (16) (p13.3)	764-3664353	3.36 Mb (detected)
2	46, XX, Del (5) (q35.3)	46, XX, Del (5) (q35.3)	178022586-180331967	2.53 Mb (detected)
3	46, XY Del (9) (p24.3)	46, XY Del (9) (p24.3)	36586-1846893	1.81 Mb (detected)
4	45, XX, Del (13) (q11q12.11)	45, XX, Del (13) (q11q12.11)	17943627-21831429	3.89 Mb (detected)
5	46, XX, Dup (6) (p25.3)	46, XX, Dup (6) (p25.3)	94648-2289621	2.19 Mb (detected)
	Del 14(q)32.12q	Del 14(q)32.12q	91720288-92918797	1.19 Mb (Not detected)

*NGS*: next generation sequencing

*aCGH*: array comparative genomic hybridization

### Multiple abnormalities on different chromosomes

**Fig. 3:**
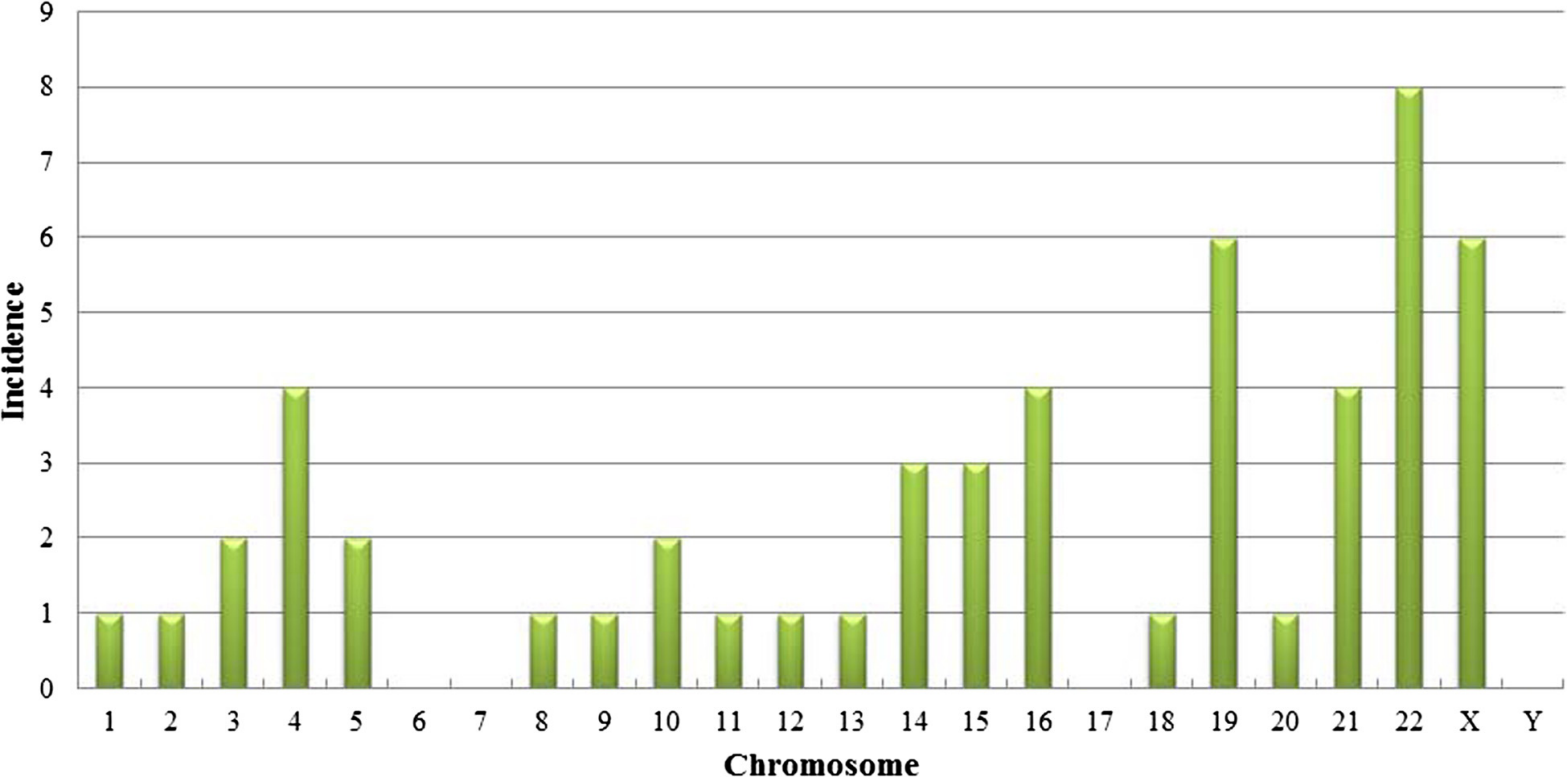
Incidence of the errors of individual chromosomes. The x-axis indicates the chromosome number, and the y-axis indicates the incidence of the chromosome errors. The abnormalities occurred to almost all chromosomes, except chromosome 6, 7, 17 and Y chromosome

Among the 41 TE cell samples with abnormal karyotypes in this study, eight (19.5 %) samples (sample # 9, 33, 34, 35, 38, 40, 41 and 43 in Table 2) presented as multiple chromosome abnormalities. The abnormalities occurred to almost all chromosomes, except chromosome 6, 7, 17 and Y chromosome. However, the incidence of abnormality was different among chromosomes (Fig. 3). Chromosome errors exceeding three times happened to eight chromosomes, and abnormality of chromosome 22 occurred up to eight times.

## Discussion

Chromosomal abnormalities may arise during germ cell and/or preimplantation embryo development. The embryonic chromosomes have direct impacts on embryo implantation and the successful development of those embryos into healthy babies. Recent years, NGS is an emerging technology that provides unprecedented high-throughput, highly parallel, and base-pair resolution data for embryo genetic analysis, but it is still under development for extensively clinical application to PGS. In the present preclinical study, we performed a validation study to determine the accuracy of an NGS-based 24-chromosome screening protocol. Trophectoderm cells from human blastocysts as well as the cytogenetically characterized cell lines with known chromosomal errors were analyzed and compared between aCGH and NGS. Our results provided a complete consistency for samples between two methods, indicating the accuracy and reliability of NGS technology for human PGS.

Clinical validation of new technologies to be applied for embryo diagnosis is particularly challenging. Previous results also showed that NGS was highly sensitive and specific for detection of aneuploidy, and segmental imbalances in 24-chromosome screening [19, 24, 25]. In addition to the validation of NGS in the detection of whole-chromosome aneuploidies in our study, cytogenetically characterized cell lines were detected meanwhile and showed that the NGS protocol was capable of accurately quantifying chromosome imbalances down to 1.8 Mb in size, indicating that diagnosis of partial aneuploidies is well within the ability of this technology.

Any strategy available for aneuploidy testing has to balance the benefits of identifying euploid embryos with the potential costs to the embryo of any invasive biopsy or any false positive and negative test results. Nowadays, largely because of efforts required to complete the Human Genome Project, DNA sequencing has undergone a steady transformation with still-ongoing developments of high-throughput sequencing machines for which the cost per reaction is falling drastically. The National Human Genome Research Institute has tracked the costs associated with DNA sequencing (available at: www.genome.gov/sequencingcost, accessed February 2015). The figure in the website showed that significant cost reduction was exhibited beginning in 2008, when sequencing centers moved from Sanger-based to NGS-based DNA sequencing technologies. The overall efficacy of PGS might be potentially further improved and eventually the advantages of NGS will be brought to PGS patients.

There are numerous advantages to using NGS for 24-chromosome aneuploidy screening. NGS-based method for copy number analysis is likely to be the most accurate and informative, because they use sequence data from thousands of loci across each chromosome. The parallel nature of NGS data provides a unique opportunity to evaluate multiple genomic loci and multiple samples on one chip. That is to say, DNA samples from different patients requiring sequence data in different genomic loci could also be evaluated on the same sequencing chip [26]. These features make NGS useful for evaluation of aneuploidy, monogene disorders and translocations simultaneously from the same biopsy without the need for multiple technological platforms [26].

Compared with aCGH, a control sample (reference gDNA sample) is not necessary for NGS. NGS does not require a sample-reference model for data normalization. A specific algorithm step is used to normalize the data and remove any bias for the sample preparation. NGS operates on a linear copy number scale instead of the logarithmic ratio scale of array experiments. Therefore, the concept of XY separation does not apply as it does for microarray.

The NGS algorithms have been developed for whole-chromosome calling. In high quality samples, it is sometimes possible to see subchromosmal imbalances. This will be possible if multiple cells are biopsied from blastocysts.

NGS has an increases dynamic range compared to 24 chromosome array and this applies to sources of noise present in the data. The main sources of noise originate from poor sample quality and amplification artifacts, including suboptimal embryo biopsy, DNA nicking, incomplete cell lysis, cells undergoing apoptosis, the presence of PCR inhibitors in media, and protocol deviations. These effects are typically more prevalent in single cell biopsy at day 3 than TE samples where greater quantities of starting materials are available. A failed amplification can be detected in most cases by observing the lack of a DNA smear in an agarose gel run as a part of the Sureplex amplification protocol.

Because the dynamic range of NGS-based PGS is higher than microarray, the copy number changes are clearly distinguishable from the normal background in good samples. It also means that any noise will be more apparent and failed samples will be easy to identify. Mosaicism is more clearly visible in NGS-based PGS.

No diagnosis (no data) in samples with aCGH can be as high as 5 % with samples from blastocyst biopsy [27], and the rate may be higher if single cells are used from day 3 blastomere biopsy [28]. As mentioned about, this may be due to many reasons, and one of the major reasons is sample quality. However, for blastocysts biopsy, multiple cells are usually separated from embryos, and greater amount of DNA would increase the amplification and reduce noises, thus improve the diagnosis rate. In the present study, we got diagnosis results from all samples tested, indicating the effectiveness of NGS-based PGS technology for samples biopsied from blastocyst.

As a general limitation, same as microarray, NGS-based PGS is not intended to detect polyploidies. Also, calling of low-level mosaicism in samples is not recommended. As with aCGH, NGS cannot directly detect balanced chromosomal rearrangements, because there is no imbalance in the total DNA content.

Other limitations include higher cost and time-consuming. Currently, NGS-based PGS cost per sample is about $10–20 more expensive than aCGH-based PGS. This may be mainly due to the early stage of this technology in human IVF-PGS. As NGS cost has been decreased significantly during the past couple of years from a few thousand dollars per sample to a few hundred dollars per sample now, it is believed that the cost will keep reducing in the future. Thus the cost may be less than aCGH-based PGS in near future.

The time for aCGH-based PGS has recently be reduced from about 20 hrs to current 8 hrs by reducing the time for DNA hybridization, this makes fresh embryo transfer to be possible if the test is on site or samples are biopsied from day 3 embryos [28]. However, NGS generally takes about 14 hrs due to time-consuming of DNA sequencing. Even so, it is still within a time frame compatible with a fresh embryo transfer if biopsy is done on day 3 and transfer is done on day 5 or day 6 [28]. However, recently, a tendency to freeze all embryos has been adopted in Northern America and other countries due to a better patient management, high embryo freeze/thawing survival rate and better embryo implantation rate per transfer, such a time frame between testing and frozen embryo transfer may not significantly limit the implementation of this technology to embryos at blastocyst stage.

## Conclusions

In conclusion, the present study was intended to be a preliminary preclinical evaluation, providing proof of feasibility for a new technology called VeriSeq NGS used for PGS. The comprehensive chromosome screening method described overcomes many of the problems that limited earlier aneuploidy screening techniques and may finally allow NGS-PGS to achieve the benefits predicted by theory. NGS can provide rapid PGS results with a high level of accuracy and more cost-effective than established methodologies in near future. Prospective clinical studies with large number of embryo biopsy specimens from patients will be implemented to obtain a larger sequencing dataset and more evidence for the extensive use of NGS-based PGS.

## Methods

### Samples and sample amplification

Forty three human embryo TE biopsy samples with known karyotype (41 abnormal and 2 normal) and 5 cytogenetically characterized cell lines (Coriell Cell Repositories) were tested. For whole genome amplification (WGA), TE cell samples and negative controls were collected in 2 μl of PBS buffer, lysed with 2 μl of SurePlex cell extraction buffer and 5 μl of the SurePlex Extraction cocktail master mix and then incubated at 75 °C for 10 min followed by further incubation at 95 °C for 4 min. Then genomic DNA (gDNA) was randomly fragmented by adding 5 μl of SurePlex Pre-amplification cocktail to the lysed biopsy samples or to gDNA controls and the mixture was incubated according to the following protocol: one cycle of 95 °C for 2 min, followed by 12 cycles of 95 °C for 15 seconds, 15 °C for 50 seconds, 25 °C for 40 seconds, 35 °C for 30 seconds, 65 °C for 40 seconds and 75 °C for 40 seconds, followed by a hold at 4 °C. Thereafter, gDNA was amplified using the PicoPLEX WGA Kit (NEB) according to the following thermal cycler program: one cycle of 95 °C for 2 min, followed by 14 cycles of 95 °C for 15 seconds, 65 °C for 1 min and 75 °C for 1 min, followed by a hold at 4 °C. To determine the success of the amplification, 5 μl of each amplified sample plus 5 μl gel loading buffer were examined by electrophoresis on a 1.5 % agarose TBE gel.

The same amplified samples were processed with both NGS protocol and aCGH protocol for aneuploidy status. Discordant samples were subsequently reevaluated by a third methodology, quantitative fluorescent polymerase chain reaction (QF-PCR), following the protocol described elsewhere [29]. When QF-PCR confirmed one of the initial methods, the remaining discordant method was considered to have delivered an erroneous result.

### NGS with VeriSeq PGS protocol

Amplified samples for NGS were processed with VeriSeq PGS kit (Illumina). DNA ‘indexing’ was performed in order to simultaneously analyze samples from different embryos, using the Nextera XT 96 - Index Kit (Illumina, Inc.). In brief, amplified samples were diluted and concentration was measured with Qubit dsDNA HS assay kit (Life technology). One nanogram total amplified DNA at 0.2 ng/ml of each sample was tagmented (tagged and fragmented) by the VeriSeq PGS transposome with manufacture’s protocol. The tagmentation step was carried out at 55 °C for 5 min and hold at 10 °C. The resulting tagmented mixture was neutralized by adding 5 ml of proprietary neutralization buffer. Post-homogenization, the Tagmentation plate was held at room temperature for 5 min.

The tagmented DNA was amplified with index primer (i7) and (i5) to become the NGS library via a limited-cycle PCR program (one cycle of 72 °C for 3 min, followed by 12 cycles of 95 °C for 10 seconds, 55 °C for 30 seconds and 72 °C for 30 seconds, one cycle at 72 °C for 30 seconds, followed by a hold at 4 °C). Each sample’s NGS library was purified with no salt carryover, providing a size selection step that removes short library fragments including index 1 (i7) and index 2 (i5) from the population. Finally 24 samples NGS library were pooled and loaded to the VeriSeq PGS (Illumina) sequencing cartridge following manufacture’s protocol. NGS library was sequenced with Illumina Miseq system. Sequencing data were generated by MiSeq Reporter Software. The following bioinformatics analysis was accomplished with a pre-release version of BlueFuse Multi for NGS (Illumina, Inc.). Each chromosome was divided into intervals each approximately covering 1 Mb of sequence. Filtered reads from each sample were then mapped into the corresponding chromosome interval or bin.

### Agilent aCGH protocol

Thirteen microlitter of amplified samples were labeled with Cy3 or Cy5 using SureTag DNA labeling kit (Agilent). Labeled samples were combined and co-precipitated with COT Human DNA in preparation for hybridization. Labelled DNA was resuspended in dextran sulphate hybridization buffer and loaded onto Agilent SurePrint G3 human CGH 8 × 60 K oligo microarrays following manufacture’s protocol. Reference DNA (both male and female were used) for array was obtained from Promega (Promega Corporation, Madison, WI USA). Thereafter, the labelled products were hybridized to array slides. After hybridization, arrays were washed to remove unbound labelled DNA and scanned with Agilent SureScan scanner at 3 μM to excite the hybridized fluorophores read and store the resulting images of the hybridization. Scanned images were analyzed by Cytogenomics 2.7.8.0 software (Agilent) following manufacture’s protocol. Human Genome Build 19 (hg19) was used in the present study. Microarray chromosome information was named by referring to Cytogenetic Nomenclatures ISCN 2013 [30].

## Abbreviations

ART: Assisted reproduction technology
IVF: In vitro fertilization
PGS: Preimplantation genetic screening
TE: Trophectoderm
aCGH: array comparative genomic hybridization
NGS: Next-generation sequencing
SNP: Single-nucleotide polymorphism microarrays
FISH: Fluorescence in situ hybridization
Q-PCR: Quantitative polymerase chain reaction
PGD: Preimplantation genetic diagnosis
WGA: Whole genome amplification
